# Re-evaluation of Incorrect Posture as a Diagnostic Criterion for Scoliosis in School Screenings: A Cross-Sectional Study in Vietnam

**DOI:** 10.7759/cureus.81535

**Published:** 2025-03-31

**Authors:** Khanh Son Hong, Pham Van Minh, Hoang Thanh Nguyen, Minh Hoang Phan, Hoai Nam Nguyen, Thu Phuong Pham

**Affiliations:** 1 Rehabilitation Department, Hanoi Medical University, Hanoi, VNM; 2 Rehabilitation Department, Ho Chi Minh City Hospital for Rehabilitation and Professional Diseases, Ho Chi Minh City, VNM; 3 Pediatrics Department, Hanoi Rehabilitation Hospital, Hanoi, VNM; 4 School of Preventive Medicine and Public Health, Hanoi Medical University, Hanoi, VNM; 5 Internal Department, Hanoi Rehabilitation Hospital, Hanoi, VNM; 6 Public Health, Faculty of Public Health, University of Medicine and Pharmacy at Ho Chi Minh City, Ho Chi Minh City, VNM

**Keywords:** adolescent, angle of trunk rotation, idiopathic scoliosis, incorrect posture, screening

## Abstract

A cross-sectional study was performed to determine the prevalence of idiopathic scoliosis through school screening and to evaluate the correlation between incorrect posture identified during screening and the confirmed diagnosis of scoliosis, utilizing the gold standard criterion in 3,527 children aged 10 to 17 years from March 2023 to December 2023 in Ho Chi Minh City, Vietnam. The evaluation method included Adam's forward bending test with the angle of trunk rotation, and a cut-off point greater than or equal to 5°. Based on clinical examination, the research results show that 312 (8.7%) children were suspected of having scoliosis. Still, the prevalence of idiopathic scoliosis confirmed by X-rays was 130 (3.6%) children, with the standard being Cobb angle ≥10°. Most people with idiopathic scoliosis had moderate curves, including 119 (91.5%) and 130 (100%) demonstrated positive vertebral body rotation, with a female-to-male ratio of 2.11:1. The study identified a correlation between suspected scoliosis and incorrect posture seen during screening, including "shoulder-height difference", "any curve in the spine", and "humps on one side" (p < 0.001). However, no correlation was determined between these listed incorrect postures and a definitive diagnosis of idiopathic scoliosis by X-rays (p > 0.05). The results of the research suggest that incorrect posture found by clinical assessment shouldn't be used as the main criterion for scoliosis diagnosis during screening. The use of the angle of trunk rotation, combined with Adam's forward bending test and a suitable cut-off angle, is required to be considered for scoliosis screening in schools.

## Introduction

Adolescent idiopathic scoliosis (AIS) is the most common form of scoliosis, affecting 2-3% of children and a female-to-male ratio of 1.5:1 to 3:1 [1,2]. The spine curvature associated with IS may advance swiftly, particularly during adolescence, leading to physical complications such as body shape deformities, alterations in thoracic volume, and cardiopulmonary function, significantly impacting aesthetics, neck and back pain, core muscle weakness, breathing problems, numbness and weakness of legs and diminishing quality of life [1,3,4]. Consequently, early detection and prompt prevention of IS are advantageous for the efficacy and treatment outcomes of patients [5].

Annually, scoliosis screening in schools is performed globally. This approach may effectively predict and diagnose adolescent spinal curvature in educational institutions while generating valuable epidemiological data on IS [6,7]. Effective scoliosis screening programs conducted by trained screening teams can help identify children with IS early, according to the Scoliosis Research Society (SRS) and the International Society on Scoliosis Orthopedic and Rehabilitation Treatment (SOSORT) [1,8]. Adam's forward bending test and angles of trunk rotation (ATR), which were evaluated with a scoliometer, are advised for children in schools by a trained screening team, specifically to detect and send those who require further study [9]. On early identification of scoliosis, children may access effective, non-surgical therapies such as braces and scoliosis-specific exercises, which may reduce the possibility of surgical intervention and serious curve development [10-12].

Scoliosis screening in Vietnam is primarily conducted through the annual children's health checkup program and relies predominantly on clinical assessment. The reported prevalence of scoliosis remains contentious due to the absence of diagnostic criteria established by the gold standard of pathology, X-rays, a technology that is both effective and safe for patients [13]. Different screening methods and unique methods may provide different outcomes [14,15]. In addition, Yan et al. (2020) revealed that incorrect posture could be a potential risk factor for scoliosis, and ATR ≥ 5° is an essential indicator for predicting the onset of scoliosis [16]. Determining local prevalence rates and implementing screening programs might substantially impact public health and alleviate the burden on the healthcare system. The lack of local data about scoliosis screening programs and their effectiveness perpetuates the debate over the need and usefulness of comprehensive screening programs, resulting in no definitive recommendations. Has screening for scoliosis in schools in Vietnam, mainly through clinical examination, correctly estimated the rate of idiopathic scoliosis in children? The hypothesis is that incorrect posture identified during clinical examination is enough for use as the main criterion for a definitive diagnosis of scoliosis. Consequently, we did scoliosis screening research in middle and high schools in Ho Chi Minh City, Vietnam, to identify children suspected of scoliosis using Adam's forward bending test added by measuring ATR with a scolimeter, and diagnosing IS through X-rays for the cohort of children suspected of scoliosis, with the goal of clarifying the correlation between disease prevalence and incorrect posture identified through clinical examination.

## Materials and methods

Setting and participants

A cross-sectional study was conducted in Ho Chi Minh City from March 2023 to December 2023. There are two distinct regions of Ho Chi Minh City, which are urban and suburban areas. In this study, two schools were selected from each area, comprising one middle school and one high school from each region. At each school, all students who met the sampling criteria were invited to participate in the study. In total, we screened 3,572 children aged 10 to 17 from four schools in Ho Chi Minh City, Vietnam, who satisfied the participation requirements and obtained parental or guardian approval. Exclusion criteria include cases involving children with congenital bone or joint disorders, those experiencing lower back pain, children missing on both occasions throughout the survey, those who refuse an X-ray when suspicion arises, or those who are absent during the screening procedure.

Study procedures

**Figure 1:**
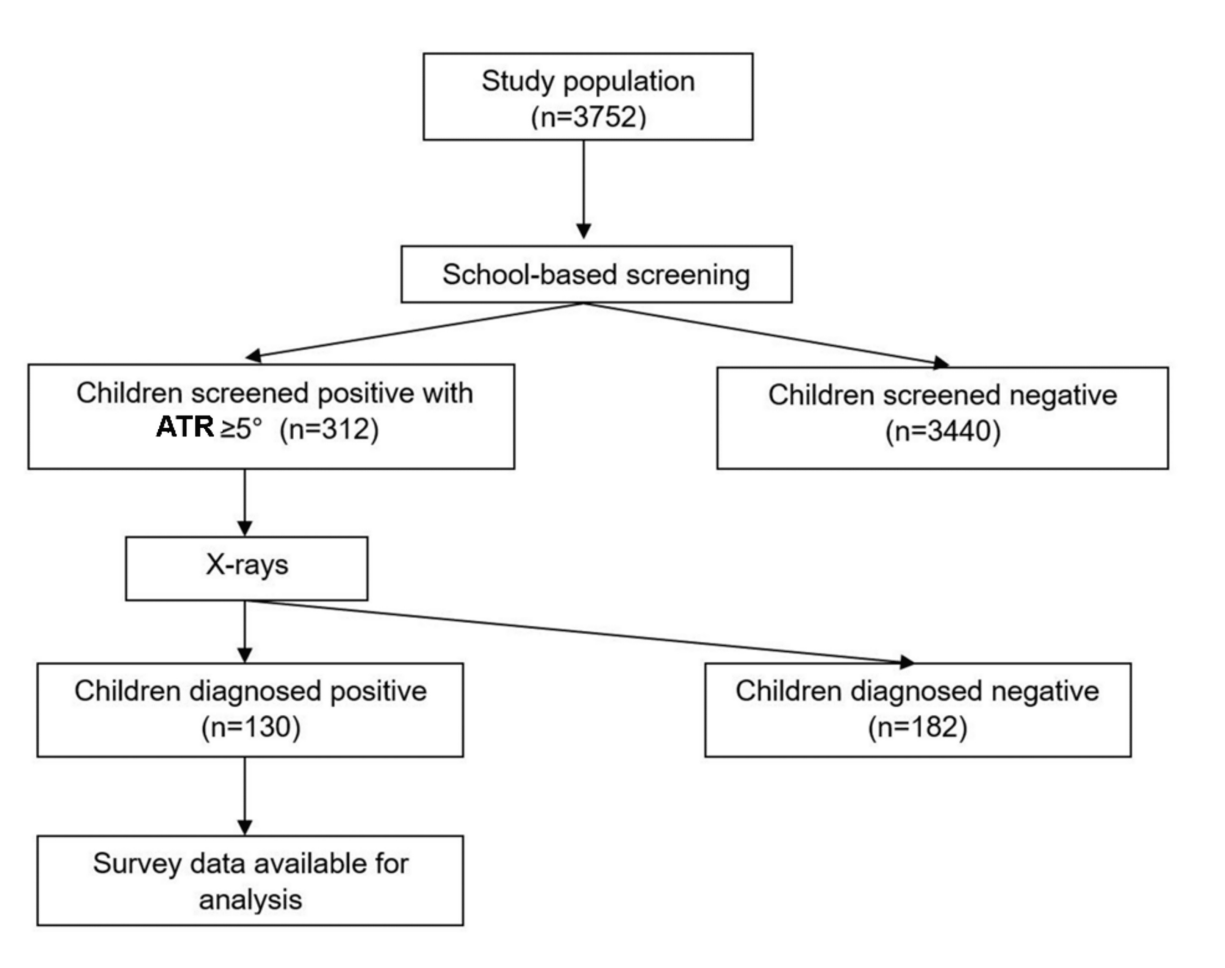
Research diagram for the screening and definitive diagnosis of AIS Every child at the school at the time of the screening and who satisfied the sample selection and exclusion criteria received a screening. Later, X-ray images of the suspected children were taken to confirm the diagnosis. AIS: Adolescent idiopathic scoliosis.

The screening program was communicated to parents by the homeroom teacher and the school where the children were studying. The researcher provides the research information sheet and permission form for study participation to the parents of children using the chosen class list. Additionally, the researcher received background information from children prior to the direct clinical assessment at the school. Children whose parents or guardians consented to participate in the research were assigned appointments on certain days of the week for clinical examination. The diagnosis of scoliosis was conducted in accordance with the study procedure. During this procedure, 3,572 children across four schools were evaluated, resulting in the identification of 312 suspected scoliosis cases. Subjects suspected of column presence were examined with X-rays for definite prediction (Figure 1).

Measurement

The data collected is derived from a pre-structured questionnaire and clinical assessment. The primary clinical measurement used for screening is the ATR, which is assessed using Adam's forward bending test with a scoliometer. The relationship between scoliometer measures and radiographic analysis was deemed strong (r = 0.7, p < 0.05), with the maximum sensitivity value recorded for ATR 5º at 87% [9]. The Cobb method is the definitive standard for assessing the angle of scoliosis on radiographs. IS is diagnosed when the Cobb angle is above 10 degrees on X-rays, and no underlying reason is discovered [5].

Furthermore, we assessed various factors associated with the incidence of suspected scoliosis during clinical examination, including the ratio of backpack weight to body weight, fashion trends, and diagnostic indicators such as sitting posture is shifted to one side, shoulder-height difference, leaning to one side, humps on one side.

Statistical analysis

Frequencies and percentages were used to describe participants’ characteristics. For quantitative variables such as age, weight, or height, means and standard deviations were used to describe the distribution. The Chi-squared test and Fisher's exact test were used to compare the prevalence of scoliosis across the participants' characteristics. Independent t-tests were used to evaluate the association between weight, height, and scoliosis. To quantify the association between scoliosis and its associated factors, odds ratios and 95% confidence intervals were calculated. A type one error rate of 5% and a 95% confidence range that excluded the value of 1 were used to indicate statistical significance. All data analyses were conducted using Stata version 17.0 (StataCorp LLC, College Station, TX).

Ethics approval and consent to participate

The present study was approved by Hanoi Medical University under Decision No. 6701/QĐ-ĐHYHN, dated December 19, 2022, and Hanoi Medical University Institutional Ethical Review Board under Decision (IRB-VN01.001/IRB00003121/FWA00004148) No. 960/GCN‑HDDDNCYSH‑DHYHN, dated August 1, 2023. The study obtained permission for collecting data from the four schools that participated in the research.

## Results

**Figure 2:**
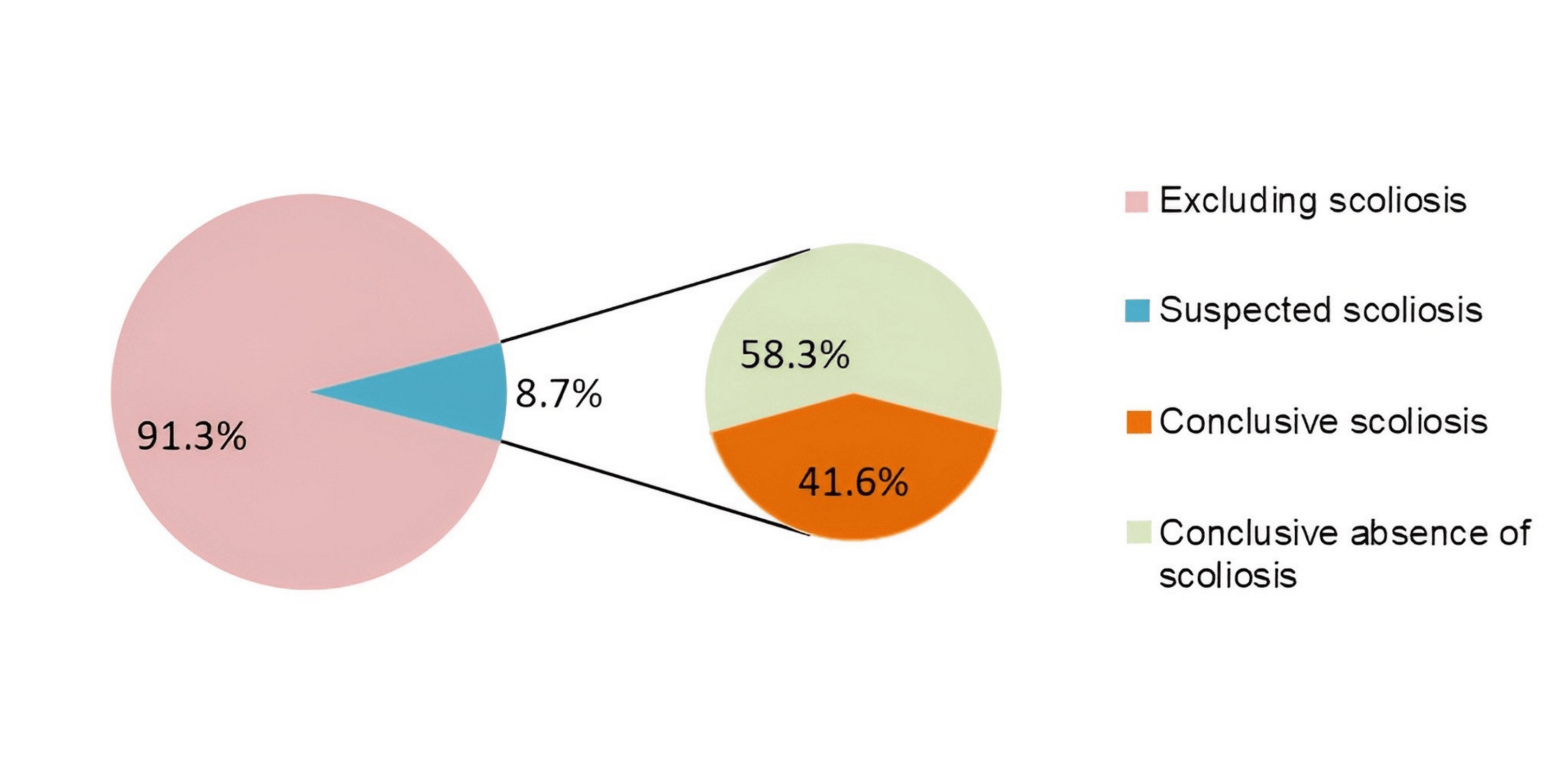
Prevalences of scoliosis by screening and definitive diagnosis X-rays were taken of patients suspected of having scoliosis following the screening examination. Participants were diagnosed with definitive scoliosis if their Cobb angle was greater than or equal to 10 degrees; the other group had a conclusive absence of scoliosis.

The study included 3,642 children, of whom 3,572 (98.1%) underwent clinical screening. The suspected case of scoliosis was identified in 312 (8.7%) of the children through clinical assessments and screenings. All children suspected of scoliosis underwent X-ray examinations. Following paraclinical examinations, the rates of children with a Cobb angle ≥ 10° and those with the conclusive absence of scoliosis pathologies were 130 (41.6%) and 182 (58.3%), respectively (Figure 2).

The prevalence of idiopathic scoliosis (IS) was shown by radiographic analysis to be 3.6% (130 of 3,572 children were available for screening). The radiographic characteristics of the patients showed that 92 (70.8%) cases had a single curve, while 38 (29.2%) presented double curves. The curvature in most IS cases was between 10 and 20 degrees, accounting for 119 (91.5%) cases. Of those with IS, 74 (56.9%) had Risser stages 3-4, and 88 (67.7%) had positive vertebral body rotation with grade (+) (Table 1).

**Table 1:** Paraclinical characteristics of idiopathic scoliosis patients (n = 130) Paraclinical characteristics observed on X-ray for patients diagnosed with definitive scoliosis having a Cobb angle ≥10°.

Characteristics	Frequency (n)	Percentage (%)
Number of curves		
1	92	70.8
2	38	29.2
Degree of curvature		
10 - 20^o^	119	91.5
21 - 40^o^	11	8.5
The Risser staging system		
Risser 0-1-2	43	33.1
Risser 3-4	74	56.9
Risser 5	13	10.0
Vertebral body rotation		
Yes	130	100
No	0	0
Grade of rotation		
+	88	67.7
++	39	30.0
+++	3	2.3

Of the 3,572 children who participated in the study, 2,565 (57.8%) chose fitted clothes as their favorite and frequently wore outfits. A significant 2,597 (72.7%) children carried schoolbag weights less than 10% of their body weight. The data collection locations include two middle schools and two high schools, with one urban and one suburban school covering each level of education. There were 1,609 (45.1%) children living in urban, and 1,963 (54.9%) in suburban, while the number of children in middle and high school was nearly comparable at 1,794 (50.4%) and 1,779 (49.6%), respectively, as shown in Table 2.

**Table 2:** Characteristics of demographic and clinical of children (n = 3572) Demographic and clinical characteristics were collected through a pre-existing questionnaire and clinical examination during the screening session. The variable “Mass of the school bag in relation to 10% of body weight” was calculated by dividing the actual weight of the child’s school bag on the day of the examination by the child’s weight.

Characteristics	Frequency (n)	Percentage (%)
Age group (years)		
11-14	1783	49.9
15-17	1789	50.1
Gender		
Male	1703	47.7
Female	1869	52.3
Weight (kg)^*^	54.0 ± 14.3	
Height (cm)^*^	156.9 ± 9.8	
Fashion trends		
Oversize clothes	1507	42.2
Fit clothes	2065	57.8
Frequently sitting on one side when studying (evaluated by teachers)		
No	2857	80.0
Yes	715	20.0
Mass of the school bag in relation to 10% of body weight		
Lower	2597	72.7
Upper	975	27.3
Residential location		
Urban areas	1609	45.1
Suburban areas	1963	54.9
School levels		
Middle school	1794	50.2
High school	1778	49.8

The study's findings in Table 3 indicate that probable scoliosis identified through screening correlates with the weight of the school bag, about 10% of body mass, among school students, and the location of data collection (p < 0.05). Children with schoolbag weights over 10% of their body mass had a 1.46-fold increased risk of developing scoliosis (95% CI: 1.14 - 1.87) compared to those with schoolbag weights below 10%. The cohort of children in suburban areas exhibited 1.69 times the risk of scoliosis relative to children in urban areas (95% CI: 1.32 - 2.16).

**Table 3:** Relationship between suspected scoliosis and their demographic, clinical characteristics (n = 3572) To determine the relationship between suspected scoliosis and non-suspected scoliosis and factors, the Chi-square test and independent t-test were used, and they were statistically significant when p<0.05.

Characteristics	Suspected scoliosis		p-value	OR (95% CI)
	Yes (n=312; 8.7%)	No (n=3260; 91.3%)		
Demographic characteristics				
Gender				
Male	141 (45.2)	1562 (47.9)	0.358^a^	1
Female	171 (54.8)	1698 (52.1)		1.12 (0.88 - 1.41)
Age group (years)				
11-14	145 (46.5)	1638 (50.2)	0.203^a^	1
15-17	167 (53.5)	1622 (49.8)		1.16 (0.92 - 1.47)
Fashion trends				
Oversize clothes	146 (46.8)	1361 (41.7)	0.085^a^	1
Fit clothes	166 (53.2)	1899 (58.3)		0.81 (0.65 - 1.03)
Weight (kg)^*^	51.1 (13.2)	54.3 (14.4)	<0.001^b^	0.98 (0.97 - 0.99)
Height (cm)^*^	156.7 (9.1)	156.9 (9.9)	0.742^b^	1.00 (0.99 - 1.01)
Mass of the school bag in relation to 10% of body weight				
Lower	204 (65.4)	2393 (73.4)	0.002^a^	1
Upper	108 (34.6)	867 (26.6)		1.46 (1.14 - 1.87)
School levels				
Middle school	151 (48.4)	1643 (50.4)	0.499^a^	1
High school	161 (51.6)	1617 (49.6)		1.08 (0.86 - 1.37)
Residential location				
Urban areas	105 (33.6)	1504 (46.1)	<0.001^a^	1
Suburban areas	207 (66.4)	1756 (53.9)		1.69 (1.32 -2.16)
Clinical characteristics				
Shoulder-height difference				
No	105 (33.7)	2689 (82.5)	<0.001^a^	1
Yes	207 (66.3)	571 (17.5)		9.28 (7.22 - 11.94)
Leaning to one side				
No	215 (68.9)	3119 (95.7)	<0.001^a^	1
Yes	97 (31.1)	141 (4.3)		9.98 (7.44 - 13.38)
Any curve in the spine				
No	178 (57.1)	3214 (98.6)	<0.001^a^	1
Yes	134 (42.9)	46 (1.4)		52.60 (36.43 - 75.95)
Humps on one side				
No	82 (26.3)	3141 (96.3)	<0.001^a^	1
Yes	230 (73.7)	119 (3.7)		74.03 (54.22 - 101.10)
Space between arms and body greater on one side				
No	206 (66.0)	3018 (92.6)	<0.001^a^	1
Yes	106 (34.0)	242 (7.4)		6.42 (4.91 - 8.39)
^*^Mean ± Standard deviation; CI: confidence interval; OR: odds ratio; ^a^Chi – square test; ^b^Independent t-test				

The research findings revealed no statistically significant connection between suspected scoliosis identified with screening and gender, age group, fashion trends, or school levels (p > 0.05). Clinical signs associated with suspected scoliosis during screening included shoulder-height difference (OR = 9.28; 95% CI: 7.22 - 11.94), leaning to one side (OR = 9.98; 95% CI: 7.44 - 13.38), any curve in the spine (OR = 52.60; 95% CI: 36.43 - 7.95), uneven contours, humps on one side (OR = 74.03; 95% CI: 54.22 - 101.10), space between arms and body greater on one side (OR = 6.42; 95% CI: 4,91 - 8.39).

We analyzed the contorted condition of 312 children suspected of having scoliosis using X-rays. The research results in Table 4 indicated a correlation between determined curvature and X-ray characteristics, including vertebral body rotation, when considering the relationship with subclinical curvature. In particular, the children with vertebral body rotation grade (++) had 3.55 times more confirmed scoliosis than those with grade (+) (95% CI: 1.52 - 7.38). The study did not identify a statistically significant relationship between the Risser staging system and curvature determined through X-rays (p > 0.05).

Concerning demographic variables, it was shown that female students had 2.11 times the probability of being diagnosed with scoliosis (95% CI: 1.33 - 3.35) in comparison to male children. Children exhibiting a fit fashion trend had 1.60 times the risk of being diagnosed with scoliosis (95% CI: 1.02 - 2.53) in comparison to those with an oversized fashion trend. Suburban children saw a 59% decrease in chances relative to urban children. The research results demonstrated no statistically significant relationship between confirmed scoliosis by X-rays and school levels, mass of the school bag, with p > 0.05. Our research findings show there is no correlation between incorrect posture such as “shoulder-height difference”, “leaning to one side”, “any curve in the spine”, “humps on one side”, “space between arms and body greater on one side” and definite scoliosis, with p > 0.05.

**Table 4:** Relationship between X-ray characteristics associated with definitive idiopathic scoliosis and clinical and demographic characteristics (n = 312). To determine the relationship between X-ray characteristics associated with definitive idiopathic scoliosis and factors, the Chi-square/Fisher’s exact test, and Independent t-test were used, and they were statistically significant when p<0.05.

Characteristics	Idiopathic scoliosis		p-value	OR (95% CI)
	Yes (n=130; 41.7%)	No (n=182; 58.3%)		
Characteristics on X-rays				
The Risser staging system				
Risser 0-1-2	43 (33.1)	77 (42.3)	0.234^a^	1
Risser 3-4	74 (56.9)	87 (47.8)		1.52 (0.94 - 2.47)
Risser 5	13 (10.0)	18 (9.9)		1.29 (0.58 - 2.89)
Grade of vertebral body rotation (n = 208)				
+	88 (67.7)	68 (87.2)	0.003^b^	1
++	39 (30.0)	9 (11.5)		3.35 (1.52 - 7.38)
+++	3 (2.3)	1 (1.3)		2.32 (0.24 - 22.78)
Demographic, social, and clinical factors				
Gender				
Male	45 (34.6)	96 (52.7)	0.002^a^	1
Female	85 (65.4)	86 (47.3)		2.11 (1.33 - 3.35)
Fashion trends				
Oversize clothes	52 (40.0)	94 (51.6)	0.042^a^	1
Fit clothes	78 (60.0)	88 (48.4)		1.60 (1.02 - 2.53)
Weight (kg)^*^	50.6 (12.7)	51.4 (13.6)	0.572^c^	1.00 (0.98 - 1.01)
Height (cm)^*^	156.8 (8.8)	156.6 (9.4)	0.880^c^	1.00 (0.98 - 1.03)
Mass of the school bag in relation to 10% of body weight				
Lower	87 (66.9)	117 (64.3)	0.629^a^	1
Upper	43 (33.1)	65 (35.7)		0.89 (0.55 - 1.43)
School levels				
Middle school	54 (41.5)	97(53.3)	0.040^a^	1
High school	76 (58.5)	85(46.7)		1.61 (0.02 - 2.53)
Residential location				
Urban areas	59 (45.4)	46 (25.3)	<0.001^a^	1
Suburban areas	74 (54.6)	136 (74.7)		0.41 (0.25 - 0.66)
Clinical clinical characteristics				
Shoulder-height difference				
No	49 (37.7)	56 (30.8)	0.202^a^	1
Yes	81 (62.3)	126 (69.2)		0.73 (0.46 - 1.18)
Leaning to one side				
No	84 (64.6)	131 (72.0)	0.166^a^	1
Yes	46 (35.4)	51 (28.0)		1.41 (0.87 - 2.28)
Any curve in the spine				
No	73 (56.2)	105 (57.7)	0.787^a^	1
Yes	57 (43.8)	77 (42.3)		1.06 (0.68 - 1.68)
Humps on one side				
No	34 (26.2)	48 (26.4)	0.965^a^	1
Yes	96 (73.8)	134 (73.6)		1.01 (0.61 - 1.69)
Space between arms and body greater on one side				
No	89 (68.5)	117 (64.3)	0.443^a^	1
Yes	41 (31.5)	65 (35.7)		0.83 (0.51 - 1.34)
^*^Mean ± Standard deviation; CI: confidence interval; OR: odds ratio;^ a^Chi – square test; ^b^Fisher’s exact test; ^c^Independent t-test				

## Discussion

Analysis findings indicated that based on clinical evaluation, 312 (8.7%) children were suspected of having scoliosis. The prevalence of IS among high school children, as determined by X-ray film diagnosis, is 130 (3.6%). This outcome diverges from prior research. Fu et al. (2024) assessed the prevalence of IS in 42 studies involving a total of 1,149,330 subjects from 30 regions by a systematic review and meta-analysis, finding rates of 1.2% [17]. Further research, including 16,045 children aged 10 to 15 years in Turkey, indicated that the screening-ready rate for ASI was 369 (2.3%), with an X-ray confirmation rate of 98.8% [18]. Research in Palermo, Italy, including children aged 11 to 14, indicated that 15.4% had IS by clinical evaluation, whereas 10.9% received a diagnosis of IS verified by Cobb angle measurement on X-rays [19]. The disparity may be attributed to variations in the screening methodology used to identify clinical indicators for diagnosing suspected scoliosis, as well as differences in age and classifications of scoliosis. In another instance, utilising ATR with a cut-off of 3°, Fok et al. determined a suspected rate of scoliosis of up to 17.8% among female children aged 10 to 13 [20]. The previously mentioned research diagnoses scoliosis by measuring the scoliosis angle using the ATR measured with a scoliometer or only by assessing clinical indicators. The authors do not use the gold standard of spinal X-rays to confirm the diagnosis after screening findings indicative of probable scoliosis. The variable incidence rates of scoliosis may be attributed to non-standardized diagnostic procedures and standards.

The paraclinical features of patients indicate that 92 (70.8%) cases demonstrate a single curve, whereas 38 (29.2%) cases have a double curve. This result closely resembles the research results of Yilmaz et al. (2020), which indicate that 69.3% of children with scoliosis exhibit a single curve, while 29.3% have a double curve [18]. The curvature range of 10 to 20 degrees constituted 119 (91.5%) cases in our study, which is consistent with other research findings. Research conducted by Fu et al. (2024) by a systematic review and meta-analysis in China showed that the curve magnitude was mainly mild (Cobb angle: 10°-19°), and the prevalence rate was 0.7%; the second was moderate (Cobb angle: 20°-39°), with a prevalence of 0.2% [17]. In accordance with the research conducted by Yılmaz et al. (2020), it was determined that 90.5% of IS cases among children had a Cobb angle ranging from 10° to 19° [18].

The analysis results indicate a correlation between IS and age (p < 0.05). Specifically, 17-year-old children have a 5.75 times higher risk of developing IS compared to 15-year-old children, with a 95% confidence interval of 2.19-15.14 and p < 0.001. This finding is consistent with the study by Zhou et al. (2023), which reported a prevalence of 3.6% in males aged 13-14, while the highest prevalence was observed in the 16-17 age group, reaching 5.2% [21]. The joint declaration of SOSORT and SRS indicates that significant advantages may be obtained by early non-invasive rehabilitation for individuals with IS. The recommendation stipulates that scoliosis screening should occur twice for females at ages 10 and 12, and once for boys at ages 13 or 14, respectively. That study's examination of suspected scoliosis prevalence by age finds that the best age for scoliosis screening in school aligns with the statement [1,8]. This discovery in Vietnam highlights the necessity of early intervention and monitoring of spinal conditions in middle school. In reality, the slow progression of initial symptoms may lead to the accumulation of spinal disorders from an early age, especially in high school tend to experience increasing physical and academic stress.

The analysis indicated a correlation among suspected scoliosis, school bag carrying method, mass of the school bag in relation to 10% of body weight, educational institution, and residential location (p < 0.05). The cohort of children with a school weight above 10% exhibited 1.46 times the probability of being diagnosed with scoliosis (95% CI: 1.14 - 1.87) (p=0.002). Nery et al. (2015) [22] showed that carrying a school bag containing 10% or more of body weight might induce back muscular dystonia, resulting in scoliosis, a conclusion supported by many other examinations documented in other studies [23]. High school children exhibited an odds ratio of 1.61 for being diagnosed with IS (95% CI from 0.02 to 2.53), paralleling the findings of Kim et al., which indicated that 10th grade children (approximately 16 years old) faced a greater risk of scoliosis compared to 7th grade children (approximately 13 years old), with incidence increasing from 1.37% in younger girls to 3.12% in older girls [24]. This indicates that while middle school children may have cases of scoliosis, the prevalence significantly increases when they transition to high school, where physical demands and development occur more quickly. The increased prevalence among high school students may possibly result from the combined effect of physical stresses and undetected spinal deformities beginning from middle school. Moreover, a significant factor is that the probability of suspecting IS among children in suburban areas is 1.69 times greater (95% CI from 1.32 to 2.16) than that of children in urban areas; however, when scoliosis is diagnosed, children in suburban areas show a 59% decreased odds compared to their urban counterparts (p < 0.001). The explanation for this finding is that children's posture in suburban regions is worse than that of those living in cities, since they show fewer concerns for their appearance and grooming. This results in a higher prevalence of suspected incorrect posture in the screening examinations for this cohort, despite a reduced percentage of verified diagnoses by X-rays. This is a crucial consideration during screenings since incorrect posture in the scapular region may lead to false-positive outcomes in evaluating vertebral rotation using Adam's test. This corresponds to the results of Zou et al., which indicated that the prevalence of positive scoliosis among children and children in urban regions was 4.1%, surpassing that in suburban areas [25].

The research results show that incorrect posture may contribute to the rising prevalence of scoliosis among children as they mature. In this investigation, we documented clinical indicators of suspicion, including shoulder-height difference, leaning to one side, any curve in the spine, humps on one side, and space between arms and body greater on one side. We observed a strong correlation with suspicion when using the main determining criteria of ATR ≥ 5° (p < 0.001). However, we found that these clinical complaints were in correlation with definitive scoliosis diagnosis (p > 0.05). This finding contrasts with the research conducted by Yan et al. (2020), which indicated that shoulder-height difference, scapula tilt, lumbar concavity, and pelvic tilt were correlated with AIS, especially in individuals exhibiting an ATR ≥ 5° in the thoracic, lumbar, and thoracolumbar regions [16]. This is a significant observation, given that research evidence indicates that using clinical indications as qualitative criteria for diagnosing scoliosis is inappropriate. The quantitative approach for measuring the ART, along with an acceptable cutoff point, should be used in conjunction with X-rays, the gold standard for diagnosing scoliosis. Even though this is a single study, more research with larger sample sizes is necessary to reconsider this matter. Additionally, inter-observer reliability for clinical posture assessments, highly susceptible to differing opinions by individuals, can be improved using quantitative indices like ART.”

Limitations of the study

When considering our findings, it is essential to consider several major limitations. At first, the causal relationship between scoliosis and the characteristics of the participants cannot be verified due to the cross-sectional design used in this study. Secondly, the study's findings are not relevant to other regions, as just two places were chosen in each of the urban and suburban areas. Lastly, the screening method based on clinical examination and Adam's forward bending test may result in errors in recognizing suspected scoliosis cases. Even though X-rays have been used to make a definitive diagnosis, there may still be mistakes in estimating and measuring the Cobb angle.

## Conclusions

In conclusion, research findings suggest a significant rise in the rate of idiopathic scoliosis identified through screening and diagnosis. Factors include female gender, high school classes, and urban location, which are risk factors that raise the possibility of scoliosis. Research suggests that incorrect posture shouldn't be used as the main criterion for scoliosis diagnosis during screening. Research suggests that incorrect posture should not be the main criterion for diagnosing scoliosis during screening. The study's findings indicate that schools should utilize trunk rotation angle measurement plus Adam's forward bending test, with an appropriate cut-off angle, such as 5°, for scoliosis screening in schools.
